# Involvement of Tyr1472 phosphorylation of NMDA receptor NR2B subunit in postherpetic neuralgia in model mice

**DOI:** 10.1186/1744-8069-8-59

**Published:** 2012-08-21

**Authors:** Sawako Unezaki, Atsushi Sasaki, Tamaki Mabuchi, Shinji Matsumura, Tayo Katano, Takanobu Nakazawa, Naoko Nishio, Tsugunobu Andoh, Tadashi Yamamoto, Terumasa Nakatsuka, Yasushi Kuraishi, Seiji Ito

**Affiliations:** 1 Department of Medical Chemistry, Kansai Medical University, Moriguchi, 570-8506, Japan; 2 Department of Applied Pharmacology, Graduate School of Medicine and Pharmaceutical Sciences, University of Toyama, Toyama, 930-0194, Japan; 3 Division of Oncology, Institute of Medical Science, The University of Tokyo, Tokyo, 108-8639, Japan; 4 Pain Research Center, Kansai University of Health Sciences, Kumatori, 590-0482, Japan

## Abstract

**Background:**

Postherpetic neuralgia is spontaneous pain and allodynia that persist long after the disappearance of the cutaneous lesions caused by herpes zoster. Inoculation of mice with herpes simplex virus-1 causes herpes zoster-like skin lesions and herpetic and postherpetic pain. Although NMDA receptors have been suggested to be involved in postherpetic pain as in other types of neuropathic pain, the neural mechanism remains unclear. NMDA receptor NR2B subunit is the most tyrosine-phosphorylated protein in the brain, and Tyr1472 is the major phosphorylation site of this subunit.

**Results:**

To elucidate the role of Tyr1472 phosphorylation of the NR2B subunit in herpetic and postherpetic allodynia, we inoculated herpes simplex virus-1 into the unilateral hind paw of knock-in mice with a mutation of Tyr1472 of the NR2B subunit to Phe (Y1472F-KI). On day 7 post-inoculation, acute herpetic allodynia was observed in more than 80% of the inoculated wild-type and Y1472F-KI mice. Y1472F-KI mice showed significantly reduced intensity and incidence of postherpetic allodynia on days 45–50 post-inoculation as compared with wild-type mice. The innervation in the skin at the postherpetic neuralgia phase was retained to a greater extent in the Y1472F-KI mice. The level of activating transcription factor-3 mRNA, a marker of axonal damage, increased much less in the dorsal root ganglia (DRGs) of Y1472F-KI mice than in those of wild-type mice; and the level of nerve growth factor mRNA significantly increased in wild-type mice, but not at all in Y1472F-KI mice on day 7 post-inoculation. Production of nerve growth factor was at the basal level in the skin of both groups of mice on day 50 post-inoculation. Nerve growth factor and glial cell-derived neurotrophic factor stimulated neurite outgrowth of cultured DRG neurons from Y1472F-KI mice, similarly or less so as they did the outgrowth of those from wild-type mice. Wild-type DRG neurons were more susceptible to glutamate neurotoxicity than Y1472F-KI ones.

**Conclusions:**

Taken together, the present data suggest that phosphorylation of the NR2B subunit at its Tyr1472 is involved in the development of postherpetic allodynia due to nerve damage and that the nerve damage at the acute herpetic phase is correlated with the incidence of postherpetic pain.

## Background

Herpes zoster, which is characterized by clustered vesicles in the skin and severe pain, is caused by the reactivation of varicella zoster virus (VZV, human herpes virus type-3), in the sensory ganglia of humans [1]. Patients with herpes zoster complain of severe spontaneous pain and allodynia, which is pain due to a non-noxious stimulus. In some herpes zoster patients, pain persists long after healing of the skin lesions, so-called postherpetic neuralgia [1]. Once established, postherpetic neuralgia is particularly difficult to treat and is often resistant to conventional analgesics. The neural mechanisms of the induction and maintenance of postherpetic neuralgia are still unclear.

Takasaki *et al.*[2,3] previously established a mouse model of herpetic and postherpetic pain by using human herpes virus 1 (herpes simplex virus type-1, HSV-1). When mice were given a transdermal HSV-1 inoculation into a hind paw, they showed herpes zoster-like skin lesions throughout the inoculated dermatome and pain-related behaviors [2]. Pain-related behaviors (herpetic allodynia) and vesicles became apparent 5 days after the inoculation, and the skin lesions healed 10 days later [2,3]. In one-third of the mice, the pain-related behaviors subsided by day 20 post inoculation; and in the rest of the mice, such behaviors (postherpetic allodynia) lasted long after the skin lesions had completely healed [3,4]. Based on data they had obtained by using this mouse model, Sasaki *et al*. [4] recently suggested that herpetic and postherpetic allodynia are mediated by nitric oxide (NO) in the dorsal horn and that inducible nitric oxide synthase (iNOS) and neuronal NOS (nNOS) are responsible for herpetic and postherpetic allodynia, respectively. Many studies including ours have demonstrated that activation of the NMDA subtype of glutamate receptors and subsequent NO production is a fundamental event in neurotransmission and synaptic plasticity in pain transmission in the spinal cord [5,6]. Garry *et al*. [7] demonstrated that the VZV-induced pain state is attenuated by intrathecal administration of an NMDA receptor antagonist and that the profile of VZV infection-induced phenotype changes in dorsal root ganglia (DRGs) are similar to those in other neuropathic pain models. We showed earlier that an increase in nNOS activity associated with Tyr1472 phosphorylation of the NR2B subunit in the superficial dorsal horn of the spinal cord reflects the neuropathic pain state even 1 week after nerve injury [8]. For elucidation of the role of Tyr1472 phosphorylation of the NR2B subunit *in vivo*, knock-in mice in which Tyr1472 of the NR2B subunit is mutated to phenylalanine (Y1472F-KI) were previously generated; and the results showed that Tyr1472 phosphorylation is critical for neuropathic pain and fear learning [9-11]. In the present study, we took advantage of this mouse model of herpetic and postherpetic pain produced by HSV-1 inoculation, in which alterations of levels of signaling molecules in the pain transmission pathway can be followed over 7 weeks in relation to pain-like behaviors *in vivo*. Applying this model to Y1472F-KI mice, we examined the role of NR2B phosphorylation in herpetic and postherpetic allodynia.

## Results

### Involvement of NR2B phosphorylation in postherpetic allodynia

**Figure 1:**
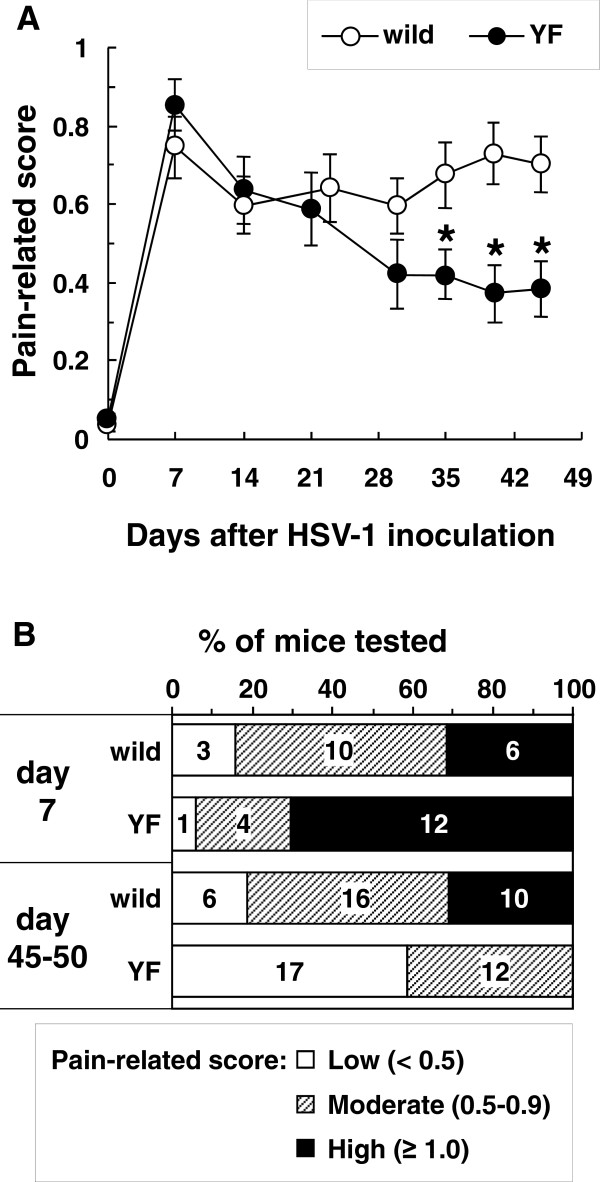
** Reduction in postherpetic neuralgia in Y1472F-KI mice.** (**A**) Time-course analysis of mechanical allodynia after HSV-1 inoculation. Wild-type and Y1472F (YF) mice were inoculated with HSV-1 on lacerated shin skin. At various times post inoculation, the plantar was stimulated by use of a von Frey filament (1.6 mN), and the response was assessed as pain-related score. Data are presented as the mean ± SEM (*n* = 19 and 17 in wild-type and Y1472F-KI mice, respectively). *P < 0.05 *vs.* wild-type mice (Mann-Whitney's *U*-test). (**B**) Comparison of the intensity of allodynia between wild-type and Y1472F-KI mice. Mice were categorized into 3 groups according to the pain-related score: low (< 0.5), moderate (0.5–0.9), and high (≥ 1.0). Data are presented as percentage of total mice tested, and the number of mice in each pain category is shown on the bars.

HSV-1 was inoculated into the shin region of C57BL/6 (wild-type) and Y1472F-KI mice to generate a model for herpetic and postherpetic allodynia. On day 7 post-inoculation, shingles were observed along the lumbar dermatome in both groups of mice (data not shown). Mechanical allodynia of the hind paw was detected in more than 80% of mice of either kind on day 7 post-inoculation, and there was no difference in the pain-related score between wild-type and Y1472F-KI mice (0.75 ± 0.08, *n* = 19 and 0.85 ± 0.06, *n* = 17, respectively; Figure 1A). Of 32 wild-type mice examined, 26 had allodynia even 7 weeks after inoculation; and 10, strong allodynia on days 45 to 50 (Figure 1B), indicating the occurrence of postherpetic allodynia. As compared with that for wild-type mice, the average pain-related score for the Y1472F-KI mice gradually decreased and became significantly lower than that for the wild-type mice by day 35 and thereafter (Figure 1A). On days 45 to 50 post-inoculation, no Y1472-KI mouse exhibited strong allodynia; and 12 of 29 Y1472-KI mice showed moderate allodynia. These results suggest that the phosphorylation of Tyr1472 in the NR2B subunit of NMDA receptors affected the development of postherpetic allodynia, but not that of acute herpetic allodynia.

### Restoration of the structure and innervation of the skin

**Figure 2:**
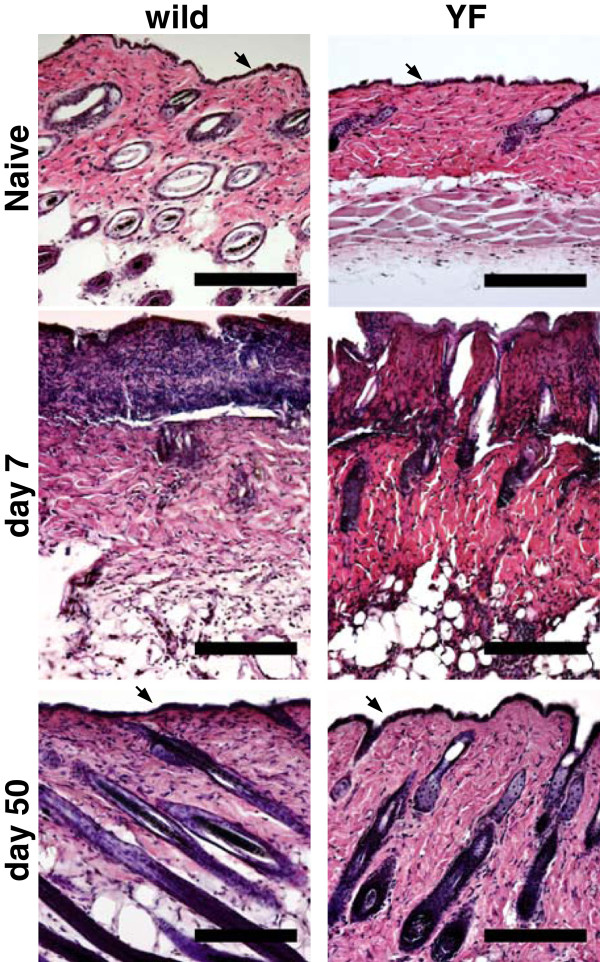
** Histological analysis of the skin of wild-type and Y1472F-KI mice.** The hindlimb skin in the lumbar dermatome was harvested from wild-type and Y1472F-KI mice before and days 7 and 50 after HSV-1 inoculation. Cross sections were stained with hematoxylin and eosin, and representative images are shown. Arrows indicate the epidermis. Scale bars = 200 μm.

Sections of the hindlimb skin of both naive wild-type and Y1472F-KI mice were examined by hematoxylin-eosin staining. The epidermal layer was observed (arrow), and hair follicles were seen in the dermal layer, in both kinds of mice; and there was no apparent difference in the skin structure between the 2 groups. The epidermal layer destroyed by shingles on day 7 post-inoculation was restored, and the skin structure with hair follicles was restored by day 50, in both wild-type and Y1472F-KI mice (Figure 2).

**Figure 3:**
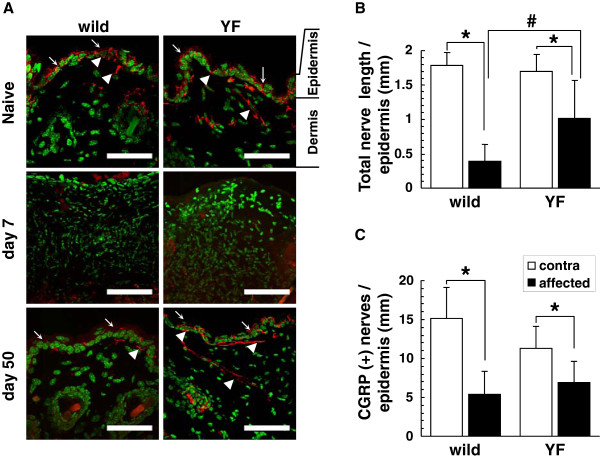
** Cutaneous innervation of wild-type and Y1472F-KI mice at postherpetic neuralgia phase.** (**A**) PGP9.5 immunostaining of nerve fibers in the skin. Cross sections of the hindlimb skin from wild-type and Y1472F-KI mice before and days 7 and 50 after inoculation were immunostained with anti-PGP9.5 antibody (red), and the nuclei were counterstained with DAPI (green). Arrows and arrowheads indicate epidermis and nerve fibers, respectively. Scale bars = 50 μm. (**B**, **C**) Total length of PGP9.5-immunoreactive nerves (**B**) and the number of CGRP-immunoreactive nerve fibers (**C**) in the epidermis. Intraepidermal nerve fibers immunoreactive for PGP9.5 and CGRP were measured in the affected skin and contralateral skin from wild-type and Y1472F-KI mice on day 50 post-inoculation, as described in the Methods section. Data are presented as the mean ± SD (*n* = 6 and 8 for PGP9.5, and 5 and 6 for CGRP, in wild-type and Y1472F-KI mice, respectively). *P < 0.05 (Bonferroni *t*-test).

Skin innervation was evaluated by immunostaining for protein gene product 9.5 (PGP9.5), a pan-neuronal marker. Nerve fibers were observed in both epidermis and dermis, and there was no difference in PGP9.5 expression between naive wild-type and Y1472F-KI mice (Figure 3A). On day 50 post-inoculation, nerve fibers were still observed in the epidermis and dermis in the wild-type mice, but their total length (0.39 ± 0.25 mm per mm width of epidermis) in the affected skin was as low as 22% of that (1.78 ± 0.18 mm) detected on the contralateral side (Figure 3B). On the other hand, the total length of nerve fibers (1.02 ± 0.55 mm) in the epidermis of the affected skin in the Y1472F-KI mice was 60% of that (1.70 ± 0.25 mm) detected on the contralateral side. Thus, the total length of nerve fibers in the affected epidermis was significantly greater in the Y1472F-KI mice than in the wild-type ones. The sensory nerve fibers in the epidermis were also examined in terms of calcitonin gene-related peptide (CGRP)-immunoreactivity. The number of CGRP-positive fibers was 5.43 ± 2.92 and 6.79 ± 2.77 fibers/mm of epidermis in wild-type and Y1472F-KI mice, respectively, being 36% and 60% of that of the contralateral side in the epidermis (Figure 3C). These results show that the innervation in the skin at the postherpetic neuralgia phase was less damaged or more restored in the Y1472F-KI mice than in the wild-type ones.

### Production of nerve growth factor (NGF) in the skin

**Figure 4:**
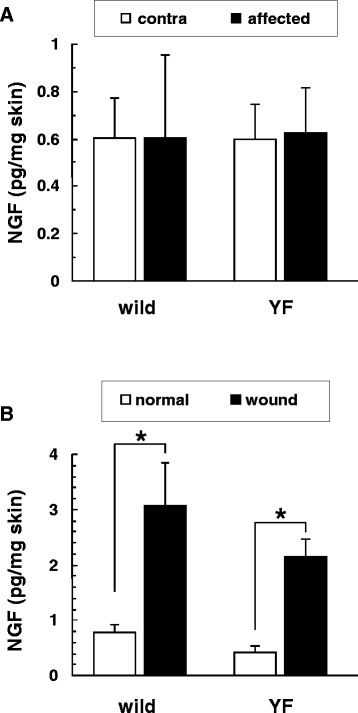
** NGF contents in the skin.** NGF contents in the skin of the affected and contralateral hindlimbs of wild-type and Y1472F-KI mice on day 50 post-inoculation (**A**) and in the back skin at 24 h after wounding (**B**) were measured by ELISA. Data are presented as the mean ± SD (*n* = 3 for wild-type, and 5 (**A**) or 3 (**B**) for Y1472F-KI mice. *P < 0.05 (Bonferroni *t*-test).

NGF, a neurotrophic factor, is produced after skin injury and stimulates nerve regeneration [12]. On day 50 post-inoculation, the NGF content in the affected skin(0.60 ± 0.34 pg/mg) of wild-type mice was not different from that in their contralateral skin (0.60 ± 0.17 pg/mg) or in the affected skin of Y1472F-KI mice (0.62 ± 0.20 pg/mg; Figure 4A). NGF production was induced after skin wounding (see Methods section for procedure): NGF contents were increased approximately 5-fold in the normal skin of wild-type and Y1472F-KI mice (3.1 ± 0.77 and 2.2 ± 0.30 pg/mg of skin, respectively; Figure 4B). Taken together, these results suggest that the NGF production in the skin had subsided after healing of shingles at the postherpetic neuralgia phase. 

### Expression of neurotrophins in DRG

**Figure 5:**
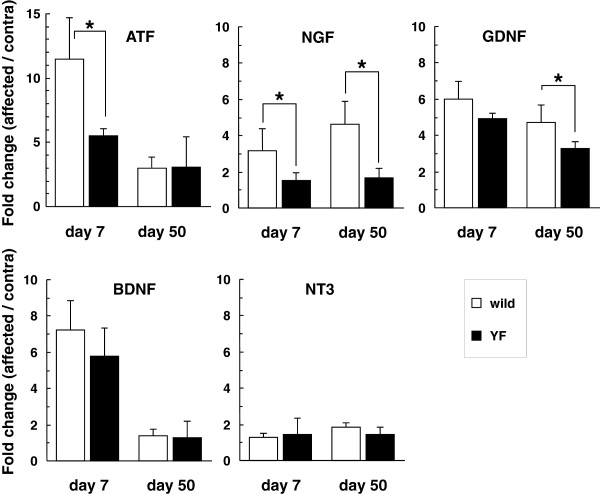
** Alterations of gene expression of neurotrophins in the DRG after HSV-1 infection.** Total RNAs were extracted from L4–L5 DRGs of wild-type and Y1472F-KI mice on days 7 and 50 post-inoculation, and cDNAs were synthesized and amplified by quantitative real-time PCR with the primers shown in the Methods section. Gene expression levels of ATF3 and neurotrophins (NGF, GDNF, BDNF, and NT3) were normalized to the GAPDH level, and are presented as the fold change in the ratio of the affected side to the contralateral one. Data show the mean ± SD (n = 3–8). * *P* < 0.05 (Bonferroni *t*-test).

To further clarify the difference in the incidence of postherpetic allodynia and the innervation of the skin between wild-type and Y1472F-KI mice, we examined the expression of activating transcription factor-3 (ATF3) and neurotrophins in the DRG on days 7 and 50 post-inoculation (Figure 5). It was earlier reported that ATF3 expression is induced in DRGs in response to nerve injury but not to demyelination and that ATF3 should thus be regarded as a neuronal marker of nerve injury [13]. The expression of ATF3 was markedly increased in the DRG on the inoculated side as compared with that in the contralateral DRG on day 7 post-inoculation, and the extent of increase was significantly lower in Y1472F-KI mice than in wild-type mice (11.5 ± 3.2 fold and 5.5 ± 0.5 fold in wild-type and Y1472F-KI mice, respectively). The increased ATF3 expression was reduced but retained on day 50 in the DRG of both wild-type and Y1472F-KI mice (3.0 ± 0.6 *vs.* 3.1 ± 2.4 fold). In contrast, NGF expression was markedly increased 3.2 fold and 4.6 fold on days 7 and 50 post-inoculation, respectively, in the wild-type DRGs, but not at all in the Y1472F-KI ones. On the other hand, brain-derived neurotrophic factor (BDNF) and glial cell-derived neurotrophic factor (GDNF) expressions were similarly increased in the affected DRGs of both wild-type and Y1472F-KI mice on day 7 post-inoculation (BDNF: 7.2 fold and 5.8 fold, respectively; GDNF: 6.0 fold and 4.9 fold, respectively). On day 50, although the increase in GDNF expression was retained in the affected DRG of both wild-type and Y1472F-KI mice (4.7 fold and 3.3 fold, respectively), the expression of BDNF had returned to its basal level by day 50. Neurotrophin 3 (NT3) expression was not increased significantly in the affected DRG of either wild-type or Y1472F-KI mice on days 7 and 50. In terms of neurotrophin receptors, the expression levels of TrkA for NGF; GFRα1, α2, and Ret for GDNF; and p75NTR were not more than twice those of the contralateral side on day 50 post-inoculation in either group of mice (data not shown). These results demonstrate that the expression of AFT3, a neuronal marker of nerve injury, was significantly low in the injured DRG of Y1472F-KI mice as compared with that in the wild-type DRG on day 7 after HSV-1 inoculation and that the NGF expression was not increased in the injured DRG of Y1472F-KI mice, either on day 7 or on day 50 after inoculation. 

### Decrease in intensity of NGF- and GDNF-sensitive fibers in the dorsal horn of the spinal cord at postherpetic neuralgia

**Figure 6:**
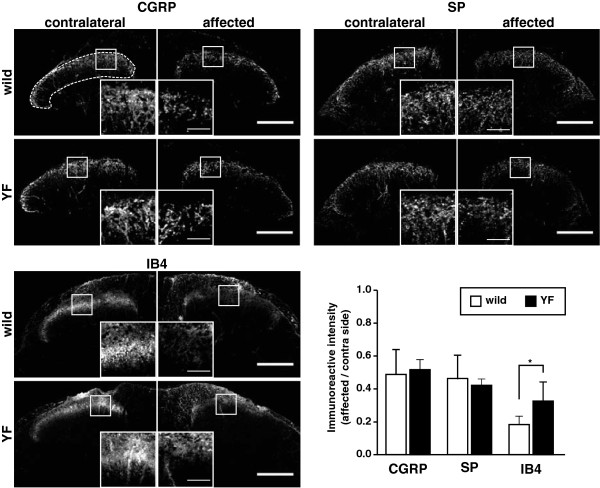
** Immunoreactivities of CGRP and SP, and IB4 labeling in the dorsal horn of spinal cord at postherpetic neuralgia phase.** Cross sections of the spinal cord of wild-type and Y1472F-KI mice at the postherpetic neuralgia phase (day 50) were immunostained with anti-CGRP and anti-SP antibodies and labeled with IB4. A representative image of the dorsal horn region of each mouse is shown, and *insets* depict higher magnification of the image delineated by the rectangles. The percentage of immunoreactive intensity in the dorsal horn (dotted line) was determined, and the data are presented as the ratio of the affected side to the contralateral one (*n* = 5). *P < 0.05 (two-tailed Student's *t*-test). Scale bars = 200 μm for low-power photos and 50 μm for high-power ones.

To examine whether the difference in the expression of NGF and GDNF in the injured DRG affected the central terminals of primary afferent fibers, we detected NGF- and GDNF-sensitive fibers by immunolabeling for CGRP and substance P (SP) and by α-D-galactosyl-binding lectin B4 (IB4), respectively, in the dorsal horn of the spinal cord on day 50 post-inoculation (Figure 6). NGF-sensitive fibers immunoreactive for CGRP and SP were decreased to 40–50% of their fluorescence intensity on the contralateral side in both wild-type and Y1472F-KI mice. GDNF-sensitive fibers labeled with IB4 were also decreased in intensity in both wild-type and Y1472F-KI mice, but the extent of the decrease was lower in Y1472F-KI mice (68%) than in the wild-type ones (82%).

**Figure 7:**
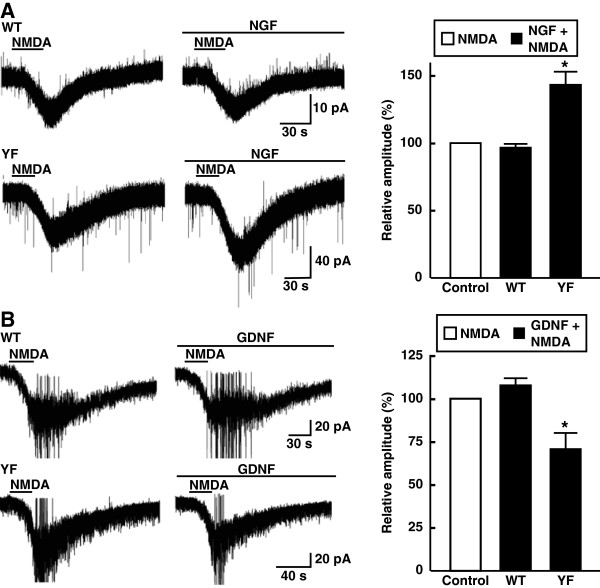
** Effects of NGF and GDNF on NMDA-induced currents in spinal SG neurons.** Representative traces of NMDA (50 μM)-induced currents in the absence and presence of 0.1 μg/ml NGF (**A**) or GDNF (**B**) in wild-type (WT) and Y1472F-KI (YF)mice are shown. The holding potentials were −40 mV. Data (mean ± SEM, *n* = 4 or 5) are expressed as relative current amplitude induced by NMDA together with NGF or GDNF to that by NMDA alone. *P < 0.05 *vs.* NMDA alone.

To examine whether NMDA receptor channels were regulated by NGF and GDNF, we measured the NMDA (50 μM)-induced currents from substantia gelatinosa (SG) neurons in the spinal dorsal horn serially in the absence and presence of 0.1 μg/ml NGF. The NMDA-induced currents were significantly increased by NGF in Y1472F-KI mice (143.6% ± 9.6%, *n* = 4) but not in wild-type mice (96.1% ± 3.6%, *n* = 5; Figure 7A). Conversely, the NMDA-induced currents were significantly attenuated by GDNF in Y1472F-KI mice (71.2% ± 9.3%, *n* = 5), but not in wild-type mice (108.3% ± 4.8%, *n* = 4; Figure 7B).

### Neurite outgrowth and neurotoxicity of DRG neurons

**Figure 8:**
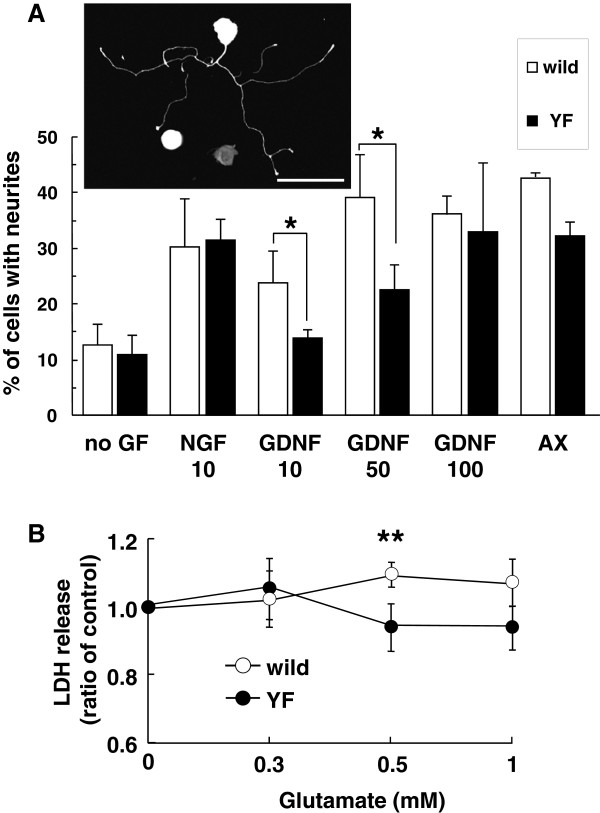
** Neurite outgrowth and neurotoxicity of DRG neurons.** (**A**) Neurite outgrowth of DRG neurons by NGF and GDNF. L4–L6 DRGs were harvested from naive or axotomized (AX) wild-type and Y1472F-KI mice at 4–5 weeks of age. DRG neurons were grown in the presence or absence of NGF (10 ng/ml) or GDNF (10, 50 or 100 ng/ml) for 22–26 h. Neurite outgrowth, shown in the photo, is presented as a percentage of total DRG cells. Data are presented as the mean ± SD (*n* = 3, 4, and 2 for NGF, GDNF, and axotomy, respectively). *P < 0.05 (two-tailed Student's *t*-test). (**B**) Neurotoxicity of glutamate toward DRG neurons. DRG neurons prepared from naive wild-type and Y1472F-KI mice were incubated without or with the indicated concentrations of glutamate for 24 h. The release of LDH was measured as described in the Methods section. Experiments (*n* = 5–6 at each concentration) were carried out by using 3 independent preparations. Data are presented as the mean ± SD of 3 experiments. *P < 0.05 *vs.* Y1472F-KI mice (two-tailed Student's *t*-test).

To further clarify whether phosphorylation of Y1472 of NR2B subunit was involved in regeneration of damaged axons or neurotoxicity, we used a DRG culture system *in vitro* to measure the neurite outgrowth and release of lactate dehydrogenase (LDH). As shown in Figure 8A, NGF at 10 ng/ml increased the neurite outgrowth of DRG neurons prepared from wild-type and Y1472F-KI mice to a similar extent (2.4- and 2.9-fold change relative to the control). On the other hand, GDNF (10 ng/ml) increased the outgrowth of neurites from the wild-type DRG (1.9 fold), but not that of those from the Y1472F-KI DRG (1.3 fold). By increasing the concentration of GDNF from 10 to 100 ng/ml, neurite outgrowth from Y1472F-KI DRG reached the same level as in the wild-type one. Axotomy of the sciatic nerve 7 days before preparation of DRG cultures increased the neurite outgrowth in DRGs from both wild-type and Y1472F-KI mice.

Finally we examined glutamate neurotoxicity toward DRG neurons by measuring LDH release. When DRG neurons were cultured with glutamate at 0.5 mM for 24 h, it did not cause the release of LDH from Y1472F-KI DRG neurons; whereas it significantly increased the release from the naive wild-type ones (Figure 8B).

## Discussion

### Involvement of Tyr1472 phosphorylation of NR2B subunit in postherpetic pain

By using wild-type and Y1472F-KI mice as nerve injury models, we recently demonstrated that phosphorylation of the NR2B subunit of the NMDA receptor is involved in neuropathic pain [10,11]. Y1472F-KI mice are born in the expected Mendelian ratios, appear healthy in terms of general behaviors, and show normal gross cytoarchitecture in their brain and spinal cord [9,10]. The expression level of NR2B and the basic properties of NMDA receptors are absolutely normal in the amygdala [9] and spinal cord [10] of Y1472F-KI mice. However, the Ca^2+^ response to glutamate is reduced after L5 spinal nerve transection injury, and the impaired NMDA receptor-mediated CaMKII signaling does not produce neuropathic pain, in Y1472F-KI mice [10]. In this study, we demonstrated by use of Y1472F-KI mice as an established mouse model of postherpetic pain that phosphorylation of the NR2B subunit affected in a positive manner the incidence of postherpetic allodynia, but not acute herpetic allodynia. In the wild-type mice inoculated with HSV-1, all mice developed mechanical allodynia; and in 81% of them the pain was sustained even after the skin shingles had healed, exhibiting the symptom of postherpetic neuralgia (Figure 1). The behavioral results are consistent with previous studies with the same animal model [3,4]. On the other hand, all Y1472F-KI mice also developed allodynia on day 7 after HSV-1 inoculation, but the pain-related score was significantly decreased after day 35; and allodynia was detected in only 41% of the mice on day 50 post-inoculation. There was no Y1472F-KI mouse with strong pain. The attenuation of postherpetic pain in Y1472F-KI mice is consistent with the results of a previous study using an NMDA receptor antagonist [7], which suggested that the NMDA receptor signaling pathway is involved in the development of postherpetic pain. Histochemical studies on adult DRG show 2 broad classes of unmyelinated C fibers: The population positive for CGRP/SP and TrkA, the high-affinity receptor for NGF, represents roughly 40% of DRG neurons in rodents; whereas the IB4-binding, GDNF-sensitive population represents roughly 30% [14]. NMDA-induced currents in the spinal cord were significantly attenuated by GDNF and enhanced by NGF in Y1472F-KI mice, but not in wild-type mice (Figure 7A). The decrease in GDNF-sensitive fibers labeled with IB4 was 68% in Y1472F-KI mice, lower than that in wild-type mice (82%), suggesting that GDNF might contribute to the reduction of intensity of postherpetic pain. Interestingly, the NGF mRNA level increased on days 7 and 50 post-inoculation only in the DRGs of wild-type mice, but not in those of Y1472F-KI mice (Figure 5). Although NGF/TrkA signals are reported to contribute to the development and maintenance of neuropathic pain [15,16], the role of NGF in the development of postherpetic pain remains unknown.

### Higher density of nerve fibers in the skin associated with reduction in postherpetic allodynia in Y1472F-KI mice

NMDA receptors located in the synaptic membranes of the spinal cord play an important role in neuropathic pain and postoperative pain due to neuronal plasticity and central sensitization [17-19]. NMDA receptors are also expressed in peripheral tissues and DRG neurons [20-22], suggesting a potential role of NMDA receptors in pain processing in the periphery [23,24]. On day 7 post-inoculation, shingles were observed along the lumbar dermatome, and the skin structure and cutaneous innervation were damaged. The skin structures recovered from these injuries on days 15–20 (not shown) [2,3], but the cutaneous innervation was markedly reduced in the wild-type mice even on day 50 (Figure 3). In Y1472F-KI mice, the degree of cutaneous innervation on the injured side also decreased, as compared with the contralateral side; but the extent of decrease was less than that on the injured side of the wild-type mice. Since cutaneous innervation was markedly decreased in the injured side of Thy1-YFP mice on day 7 after inoculation ( Additional File 1: Figure S1), the disappearance of PAG9.5 immunoreactivity in the skin on day 7 (Figure 3) was due to the loss of nerve fibers rather than the alteration in immunoreactivity. It has been reported that cutaneous innervation is decreased in patients with postherpetic neuralgia and that intraepidermal nerve fiber density is negatively correlated with the severity of pain in patients with herpes zoster [25-27] as well as in those with other sensory neuropathies and in several animal models [28-30]. In fact, it was reported that excision of painful skin relieved long-lasting postherpetic pain in a patient [31].

### Reduced damage to peripheral nerve results in reduced postherpetic allodynia

DRG neurons contain NR1, NR2B, NR2C, and NR2D, but not NR2A, subunits [20]. Furthermore, NR2B subunits are predominantly expressed on small-diameter primary afferents [21]. There are 2 possibilities for higher density of nerve fibers in the skin of Y1472F-KI mice (Figure 3): reduced damage to nerve fibers and better regeneration of lost nerve fibers in the skin. The findings in the present study demonstrate that Tyr1472 phosphorylation of NR2B subunit was involved in the damage to nerve fibers for the following reasons: (1) When herpes virus (HSV-1 or VZV-infected cells) is inoculated transdermally, the viral DNA and the gene products are detected in the DRGs and alter the gene expression of neuropeptides and ion channels in these ganglia [2,7]. ATF3 is highly induced in all injured DRG neurons after peripheral nerve injury, and the increase in the number of ATF3-positive DRG neurons is correlated positively with pain behavior [32]. The ATF3 expression level was higher on day 7 post-inoculation in wild-type mice than in Y1472F-KI mice, and the increased expression was still retained on day 50 (Figure 5), indicating that axonal damage was more severe in the skin of wild-type mice than in that of Y1472F-KI mice. Similarly, GDNF-sensitive neurons labeled with IB4 in the spinal dorsal horn were higher in number in Y1472F-KI mice than in wild-type mice (Figure 6). (2) It has been reported that the NGF level in the DRG increases in other neuropathic pain model animals [33,34] and that NGF/TrkA signals contribute to the development and maintenance of neuropathic pain [35,36]. Although the NGF level was previously reported to increase in the injured skin [12,37], it had returned to the basal level on day 50 after the HSV-1 injection (Figure 4). Therefore it is unlikely that NGF directly activated and/or sensitized the nociceptors on free nerve endings at the postherpetic pain phase. (3) The neurite outgrowth from adult DRGs prepared from Y1472F-KI mice was similarly facilitated by NGF, but the responsiveness of these mice to GDNF was reduced as compared with that of wild-type mice to it (Figure 8A). On the other hand, LDH release, indicative of cell damage, was observed in wild-type DRG neurons treated with glutamate at 0.5 mM, whereas no release was observed in the case of the Y1472F-KI DRG (Figure 8B). Taken together, these results demonstrate that reduced nerve damage by HSV-1 in eruptions lowered the incidence of postherpetic neuralgia. This notion is consistent with earlier findings that the inhibition of acute herpetic allodynia results in a decreased incidence of delayed postherpetic allodynia [38].

## Conclusions

The application of the postherpetic pain model by HSV-1 inoculation into Y1472F-KI mice is useful for examining the factors contributing to the transition from acute herpetic pain to delayed postherpetic pain. Phosphorylation of the NR2B subunit was involved in the pathogenesis of neuropathic pain not only in the spinal cord but also in that of postherpetic pain in the periphery.

## Methods

### Animals

Animal experiments were approved by the Animal Experimentation Committee of Kansai Medical University and the Animal Care Committee at University of Toyama, and were carried out in accordance with the National Institutes of Health Guide for the Care and Use of Laboratory Animals. C57BL/6 mice were obtained from Japan SLC (Shizuoka, Japan). Y1472F-KI mice were generated by a knock-in technique, substituting Tyr1472 (Y) of NR2B subunit with Phe (F). The absence of Tyr1472 phosphorylation was confirmed, and the mice were backcrossed to the C57BL/6 J background as described previously [9]. Eight- to 10-week old mice were used for HSV-1 inoculation.

### HSV-1 inoculation

Mice were inoculated with HSV-1 as described previously [2]. Briefly, HSV-1 (7401 H strain; 1 × 10^6^ plaque-forming units in 10 *μ*l) was inoculated onto the shin skin on the lumbar dermatome of the right hind paw after scarification with 27-gauge needles. The contralateral hind paw was not inoculated.

### Assessment of allodynia

Punctate allodynia of the hind paw was assessed as described previously [4]. Briefly, mice were acclimated to the instrument, and then their plantar skin was pressed by a von Frey filament having a bending force of 1.6 mN (ca. 0.16 g). The responses to the stimulus were ranked as follows: 0, no response; 1, lifting the hind paw; 2, flinching or licking the hind paw. The stimulation was applied 6 times, and the average served as the pain-related score. Mice that showed 0.5 or higher as their pain-related score were considered to have allodynia; and those with ≥ 1.0, to have strong allodynia.

### Histology

On day 7 or 50 after the inoculation, the mice were anesthetized with 50 mg/kg of pentobarbital and perfused transcardially with a fixative solution (4% paraformaldehyde in 0.1 M phosphate buffer, pH 7.4). The hindlimb skin on the lumbar dermatome was harvested from both inoculated and contralateral sides. The lumbar spinal cord was also harvested from the mice on day 50 post-inoculation. Tissues were post-fixed by immersion in the above-mentioned fixative solution at 4°C overnight, and then incubated in 30% sucrose in phosphate-buffered saline (PBS) overnight. Tissues were frozen in Tissue Mount® (Chiba Medical, Saitama, Japan) or O.C.T. compound (Sakura Finetek, Tokyo, Japan) and cut transversely into 10- or 20-μm sections on a cryostat. The skin sections were stained with hematoxylin and eosin, and images were captured with a color cooled charge-coupled device camera mounted on a microscope (E1000; Nikon, Tokyo, Japan).

### Immunohistochemistry

Tissue sections were first incubated for 30 min in a blocking solution (5% normal goat serum and 0.25% Triton-X 100 in PBS) and then incubated with rat monoclonal anti-SP antibody (1:100; AbD Serotec, Oxford, UK) or rabbit polyclonal antibodies against PGP9.5 (1:500; Abcam, Cambridge, UK) or CGRP (1:4000, Sigma, St. Louis, MO), overnight at 4°C. After having been washed with PBS, the sections were incubated with Alexa 488-conjugated goat anti-rat or Alexa 546-conjugated goat anti-rabbit IgG antibody (1:500; Invitrogen, Carlsbad, CA) for 1 h, and washed. The sections were then counterstained and mounted with coverslips by using Vectashield mounting medium containing 4',6-diamidino-2-phenylindole (DAPI; Vector Laboratories, Burlingame, CA). The fluorescent images were obtained with a Zeiss laser scanning confocal microscope (LSM 510 META; Carl Zeiss, Jena, Germany). A stack of images of tissue sections was created from a series of 34–48 consecutive images taken along the z-axis, each having a 0.38-μm thickness, with Zeiss C-Apochromat 40 x/1.2 W.

### IB4 labeling

Sections of spinal cords were preincubated for 20 min at room temperature with 0.1% Triton-X 100 in PBS supplemented with 0.1 mM CaCl_2_, MgCl_2_, and MnCl_2_ and then incubated overnight at 4°C with 50 *μ*g/ml of fluorescein isothiocyanate-conjugated *Bandeiraea simplicifolia* isolectin B4 (IB4, Sigma) in the preincubation buffer.

### Quantitative image analysis

All image analyses were conducted with ImageJ software (http://rsbweb.nih.gov/ij/). Quantification of epidermal nerve fibers immunoreactive for PGP9.5 and CGRP was done by using skin sections on day 50 post-inoculation. As for PGP9.5-positive epidermal nerve fibers, 8 to 10 photos of the affected skin and 5 or 6 photos of the contralateral skin were taken at regular intervals per section per mouse.

The lengths of PGP9.5-positive intraepidermal nerve fibers were measured with ImageJ software, and the total length (per mm of epidermis) of all nerve fibers in each image was calculated. The number of CGRP-positive nerve fibers in the epidermis was counted in 3 sections per mouse at a magnification of 630. The mean numbers of nerve fibers per mm epidermis were calculated. For measurement of CGRP-, SP-, and IB4-fluorescence intensity in the spinal cord, every 6 sections of the dorsal horn were analyzed per mouse. The fluorescent images were converted to binary images, and the area of labeled neurons was measured.

### Enzyme-linked immunosorbent assay (ELISA)

On day 50 post-inoculation, the hair of mice was clipped; and the hindlimb skin of both inoculated and contralateral sides was harvested. A skin-wound model was made as previously described [37]. Briefly, adult C57BL/6 and Y1472F-KI mice were anesthetized by pentobarbital, after which the hair on their back was clipped and full-thickness skin wounds 5 mm in diameter were prepared by cutting out the skin. After 24 h, the skin approx. 2–3 mm around the wound was obtained and stored at −80°C. The frozen skin was ground into a powder in a mortar chilled by liquid nitrogen, and the skin powder was weighed. The powder was then suspended in lysis buffer (20 mM Tris, 137 mM NaCl, 1% NP40, 10% glycerol, 1 mM phenylmethylsulfonyl fluoride, 10 *μ*g/ml trasirol; pH 8), and the mixture was subsequently stirred for 30 min on ice to extract NGF. After centrifugation at 15,000 rpm for 20 min, the supernatant was recovered; and the NGF content was then measured by use of an ELISA kit (Promega, Madison, WI) according to the manufacturer’s protocol.

### PCR analysis

Total RNAs were extracted from L4 and L5 DRGs of wild-type and Y1472F-KI mice on days 7 and 50 post-inoculation by using Trizol reagent as recommended by the manufacturer (Invitrogen). cDNAs were synthesized from the RNA by use of reverse transcriptase with p(dN)_6_ random primer. Real-time PCR was carried out by using an MJ Research Opticon 2 system (BioRad, Hercules, CA) with SYBR green and the following primers (5'-to-3' direction): aagactggagcaaaatgatg and gacttggtgactgacatctc for ATF3, cagacactctggatctagac and gattggaggctcggcacttg for NGF, cgttgagaaagctgcttcag and tcggctttgctcagtggatc for BDNF, ctgtctgcctggtgttgct and cgtcatcaaactggtcagga for GDNF, gagactgaatgaccgaactc and tatccgcctggatcagcttg for NT3, and ttgggcgcctggtcaccagggctgc and atttgccgtgagtggagtcatac for GAPDH. Each sample was analyzed in triplicate, and the data were normalized to GAPDH by using the comparative C_t_ method.

### Electrophysiology

The methods used for obtaining spinal cord slice preparations were described previously [39]. In brief, adult wild-type and Y1472F-KI mice (8–10 weeks of age) were deeply anesthetized by an intraperitoneal injection of urethane (1.2 g/kg), and then lumbosacral laminectomy was performed. The lumbosacral spinal cord (L1–S3) was removed and placed in preoxygenated Krebs’ solution at 1–3°C. Immediately after the removal of the spinal cord, the mice were given an overdose of urethane and then killed by exsanguination. The pia-arachnoid membranes were removed after cutting all the ventral and dorsal roots near the root entry zone. The spinal cord was mounted on a microslicer, and then a 650-mm-thick transverse slice was cut. The slice was placed on a nylon mesh in the recording chamber, which had a volume of 0.5 ml, and was then perfused at a rate of 10–15 ml/min with Krebs’ solution saturated with 95% O_2_ and 5% CO_2_ and maintained at 36 ± 1°C. The Krebs’ solution contained 117 mM NaCl, 3.6 mM KCl, 2.5 mM CaCl_2_, 1.2 mM MgCl_2_, 1.2 mM NaH_2_PO_4_, 25 mM NaHCO_3_, and 11 mM glucose (pH = 7.4).

Blind whole-cell patch-clamp recordings were made from substantia gelatinosa (SG) neurons with patch-pipette electrodes having a resistance of 5–10 MΩ [39]. The patch-pipette solution was composed of 135 mM potassium gluconate, 5 mM KCl, 0.5 mM CaCl_2_, 2 mM MgCl_2_, 5 mM EGTA, 5 mM HEPES, and 5 mM ATP-Mg (pH = 7.2). Signals were acquired with a patch-clamp amplifier (Axopatch 200B; Molecular Devices, Sunnyvale, CA). Data were digitized with an A/D converter (Digidata 1440A, Molecular Devices), stored, and analyzed with a personal computer using the pCLAMP data acquisition program (Version 10.2, Molecular Devices). SG neurons were viable for up to 24 h in slices perfused with preoxygenated Krebs’ solution. However, all the recordings described here were obtained within 12 h. Whole-cell patch-clamp recordings were stable for up to 4 h. Drugs were dissolved in Krebs’ solution and applied by perfusion via a 3-way stopcock without any change in the perfusion rate or the temperature. The time necessary for the solution to flow from the stopcock to the surface of the spinal cord slice was approximately 10 sec.

### DRG culture

L4, L5, and L6 DRGs were dissected from C57BL/6 and Y1472F-KI mice at 4 to 5 weeks of age. Some mice were subjected to a unilateral axotomy. The left sciatic nerve was ligated and cut at mid-thigh level under pentobarbital anesthesia. Seven days later, the animals were killed; and their ganglia were then collected. The ganglia were digested with 0.7 mg/ml collagenase type II (Sigma) in Dulbecco's modified Eagle medium (DMEM) for 50 min at 37°C followed by 0.05% trypsin-EDTA for 5 min at 37°C, and then the digestion was stopped by the addition of 0.2 mg/ml of trypsin inhibitor (Sigma). The cells were dispersed by pipetting and then filtered through a 70-*μ*m cell strainer (BD). The cell number was counted, and the cells were plated onto poly-D-lysine and laminin-coated glass cover slips (in 24-well plates) at a density of 2.5 × 10^3^ cells/ml and cultured in Neurobasal medium with 2% B27 supplement. To some wells 10–100 ng/ml of NGF or GDNF (PeproTech, Rocky Hill, NJ) was added, and the cells were then cultured at 37°C in a humidified atmosphere containing 5% CO_2_. After 22–26 h of culture, the neurons were fixed in 4% paraformaldehyde for 15 min on ice and washed with PBS. Non-specific binding sites were blocked with blocking solution (2% bovine serum albumin, 0.25% Triton X-100 in PBS) for 30 min at room temperature, and then the fixed cells were incubated overnight at 4°C with anti-PGP9.5 antibody (Ultraclone) diluted 1:1000 in blocking solution. After having been washed with PBS, the neurons were incubated with a 1:500 dilution of Alexa 546-conjugated goat anti-rabbit IgG (Invitrogen) for 1 h at room temperature and washed in PBS. After the coverslips had been mounted to glass slides with Vectashield (Vector Laboratories), the immunostained neurons were observed with a Zeiss laser scanning confocal microscope (LSM 510 META, Carl Zeiss); and 200–400 cells per sample were counted to measure the percentage of cells with neurite outgrowth. The cells that extended neurites to a length greater than 2 cell-body diameters were classified as process-bearing neurons.

### Release of lactate dehydrogenase (LDH)

LDH release was measured with an LDH-Cytotoxic Test kit (Wako Pure Chemicals, Osaka, Japan) according to manufacturer's instructions. Briefly, after DRG neurons (3,000/well) had been incubated without or with 0.1–1 mM glutamate in a 96-well plate for 24 h, 50 μl of culture medium was transferred from each well to a well of another 96-well plate; and 50 μl of freshly prepared coloring solution was added to it. LDH activity was colorimetrically measured at 560 nm by use of a plate reader (Enspire 2300 Multilabel Reader, PerkinElmer, Waltham, MA).

### Statistics

Data were presented as mean ± standard deviation (SD). The Mann–Whitney *U*-test was used for the statistical analysis of allodynia data, and Bonferroni *t*-test or Student's *t*-test (two-tailed) was used for other analyses, by using SigmaPlot ver. 12 (Systat Software, Inc., San Jose, CA). *P* values of less than 0.05 were considered significant.

## Competing interests

The authors declare that we have no competing interests.

## Authors’ contributions

SU and TM were involved in data acquisition for most experiments except for those involving behaviors; and AS, TA, and YK, in the preparation and behavioral analysis of the postherpetic pain model. SM and TK participated in the *in vitro* experiments using DRGs. NN and TN were involved in the electrophysiological studies. TN and TY generated the Y1472F-KI mice. SI is the corresponding author and participated in the design of experiments and manuscript preparation. All authors read and approved the final manuscript.

## Supplementary Material

Additional file 1 ** Figure S1.** Cutaneous innervation in Thy1-YFP mice on day 7 after inoculation. Thy1-YFP transgenic mice were obtained from Jackson Laboratory (strain B6.Cg-Tg (thy1 YFP) 16Jrs/J). Ten-week-old mice, weighing 20 ± 2 g, were inoculated with HSV-1 and the hindlimb skin in the lumbar dermatome was harvested from the mice on day 7 after the inoculation. The fluorescent images were obtained with a Zeiss laser scanning confocal microscope (LSM 510 META; Carl Zeiss, Jena, Germany). Scale bar = 50 μm. Click here for file
