# Supplementary material for: Involvement of Tyr1472 phosphorylation of NMDA receptor NR2B subunit in postherpetic neuralgia in model mice
Source: Mol Pain. 2012 Aug 21;8:59. doi: 10.1186/1744-8069-8-59 (PMC3495680; doi:10.1186/1744-8069-8-59)
Supplement: Additional file 1 — Figure S1. Cutaneous innervation in Thy1-YFP mice on day 7 after inoculation. Thy1-YFP transgenic mice were obtained from Jackson Laboratory (strain B6.Cg-Tg (thy1 YFP) 16Jrs/J). Ten-week-old mice, weighing 20 ± 2 g, were inoculated with HSV-1 and the hindlimb skin in the lumbar dermatome was harvested from the mice on day 7 after the inoculation. The fluorescent images were obtained with a Zeiss laser scanning confocal microscope (LSM 510 META; Carl Zeiss, Jena, Germany). Scale bar = 50 μm. [file 1744-8069-8-59-S1.doc]

**Additional file**

**
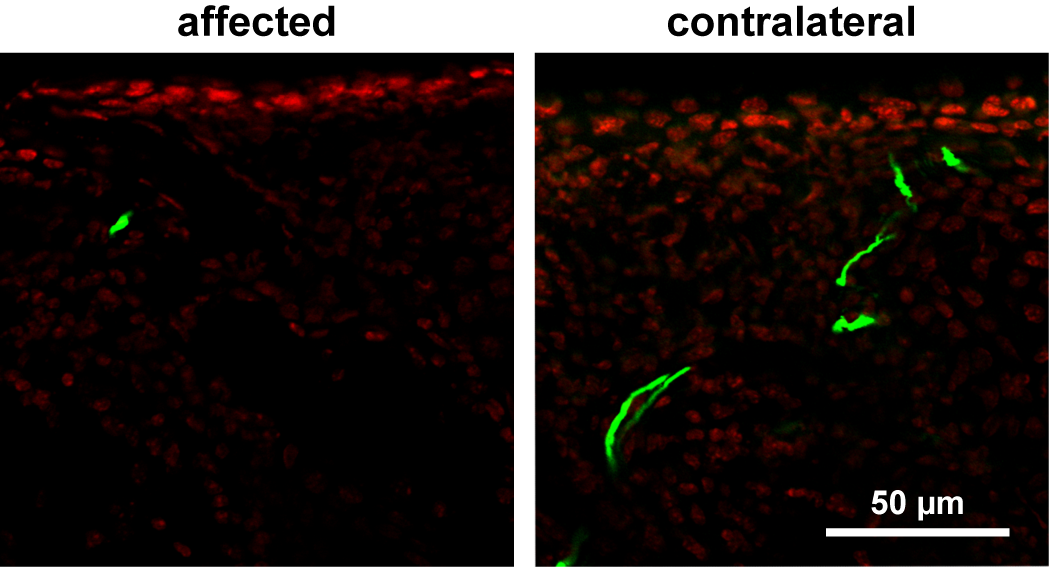
**

**Additional file 1** Cutaneous innervation in Thy1-YFP mice on day 7 after inoculation.

Thy1-YFP transgenic mice were obtained from Jackson Laboratory (strain B6.Cg-Tg (thy1 YFP) 16Jrs/J). Ten-week-old mice, weighing 20±2 g, were inoculated with HSV-1 and the hindlimb skin in the lumbar dermatome was harvested from the mice on day 7 after the inoculation. The fluorescent images were obtained with a Zeiss laser scanning confocal microscope (LSM 510 META; Carl Zeiss, Jena, Germany). Scale bar = 50 μm.
